# Four key challenges in the open‐data revolution

**DOI:** 10.1111/1365-2656.13567

**Published:** 2021-09-15

**Authors:** Roberto Salguero‐Gómez, John Jackson, Samuel J. L. Gascoigne

**Affiliations:** ^1^ Department of Zoology University of Oxford Oxford UK; ^2^ Max Planck Institute for Demographic Research Rostock Germany

**Keywords:** big data, demography, FAIR, individual‐level data, interoperability, open access, ornithology, reproducible research

## Abstract

**In Focus:** Culina, A., Adriaensen, F., Bailey, L. D., et al. (2021) Connecting the data landscape of long‐term ecological studies: The SPI‐Birds data hub. *Journal of Animal Ecology*, https://doi.org/10.1111/1365‐2656.13388. Long‐term, individual‐based datasets have been at the core of many key discoveries in ecology, and calls for the collection, curation and release of these kinds of ecological data are contributing to a flourishing open‐data revolution in ecology. Birds, in particular, have been the focus of international research for decades, resulting in a number of uniquely long‐term studies, but accessing these datasets has been historically challenging. Culina et al. (2021) introduce an online repository of individual‐level, long‐term bird records with ancillary data (e.g. genetics), which will enable key ecological questions to be answered on a global scale. As well as these opportunities, however, we argue that the ongoing open‐data revolution comes with four key challenges relating to the (1) harmonisation of, (2) biases in, (3) expertise in and (4) communication of, open ecological data. Here, we discuss these challenges and how key efforts such as those by Culina et al. are using FAIR (Findable, Accessible, Interoperable and Reproducible) principles to overcome them. The open‐data revolution will undoubtedly reshape our understanding of ecology, but with it the ecological community has a responsibility to ensure this revolution is ethical and effective.

## Graphical abstract

Following numerous calls to archive the world's biodiversity before we continue to lose it, open‐access databases of ecological data continue to grow both in number and volume. The latest contribution to the collective efforts is the SPI‐Birds data hub, which contains long‐term, individual‐level records for multiple bird species as well as ancillary data (e.g. genetics). Here the authors highlight how efforts such as SPI‐Birds are contributing to overcome challenges in open access in ecology.

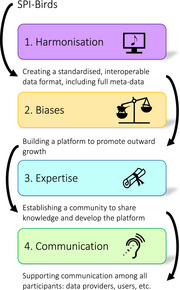

## Sumario

**Enfocado:** Culina, A., Adriaensen, F., Bailey, L. D., et al. (2021) Connecting the data landscape of long‐term ecological studies: the SPI‐Birds data hub. *Journal of Animal Ecology*, https://doi.org/10.1111/1365‐2656.13388. La información a largo plazo y a nivel de individuo ha cementado numerosos descubrimientos clave en la ecología, y las llamadas para la recopilación, conservación, y publicación de este tipo de datos ecológicos están contribuyendo a una revolución de información abierta en la ecología. Las aves, en particular, han sido el foco de la investigación internacional durante décadas, el cual ha resultado en una serie de estudios únicos a largo plazo. No obstante, historicamente el acceso libre a esta información ha representado un desafío importante. Culina y colegas (2021) presentan un repositorio online de registros de aves a nivel individual y de alta replicación temporal con metadatos (por ejemplo, genética) que permitirá explorar importantes preguntas ecológicas a grandes escalas espaciales. Sin embargo, además de las oportunidades presentadas en esta base de datos, argumentamos que la revolución de la información abierta viene con cuatro desafíos clave relacionados con (1) la armonización de, (2) los sesgos en, (3) la experiencia en y (4) la comunicación de información ecológica de forma abierta y transparente. Aquí discutimos estos desafíos y cómo esfuerzos clave como los de Culina y colaboradores están utilizando los principios FAIR (por sus siglas en inglés: Localizable, Accesible, Interoperable y Reproducible) para superarlos. La revolución de la información abierta sin duda remodelará nuestro entendimiento de la ecología. Sin embargo, la comunidad ecológica tiene la responsabilidad de garantizar que esta revolución sea ética y eficaz.

It has been over a decade since the publication of Clutton‐Brock and Sheldon's (2010) review highlighting the importance of long‐term, individual‐based studies of animals for our understanding of ecology. The importance of these studies has in no way lessened. Key discoveries from long‐term, individual‐based studies in the last decade have been made on topics as broad as the evolution of sociality (Aplin et al., 2015; Firth et al., 2018), the role of climatic variation on intra‐annual population dynamics (Paniw et al., 2019) and the role of individual differences in shaping ecological interactions (Griffiths et al., 2020). However, a new era in ecological research has emerged—the era of open data. Here, opportunities lie not only in the temporally rich insights made by one study but also in the broad patterns revealed by many. The recent publication in the *Journal of Animal Ecology* by Culina et al. (2021) introducing the SPI‐birds data hub is an important contribution towards the ongoing momentum that is bringing ecology into this new era: one where data, tools, pipelines and expertise/advise are shared unconditionally and for free across the community.

Calls to arms to ecologists for a more biogeographically representative, longer‐term, open‐access body of biodiversity data are not new. However, these calls have become more prominent in recent years (Mills et al., 2015; Wilson, 2017). Recognition of the importance of open‐access data and reproducible research pipelines in ecology has led multiple funding agencies (e.g. NERC, NSF, ARC) and journal publishers, including the British Ecological Society (2016), to ‘strongly suggest’ in the first instance, and later to mandatorily require for published research to be FAIR (Wilkinson et al., 2016): Findable, Accessible, Interoperable (i.e. data can interact with other data and workflows) and Reusable. Precipitated by this new research model, but also by ecologists' ethos regarding open access (Gallagher et al., 2020), volumes of ecologically relevant data are being amassed and subsequently released; these titanic efforts continue despite the glaring lack of funding support in most countries (Farley et al., 2018; Hampton et al., 2013).

**FIGURE 1:**
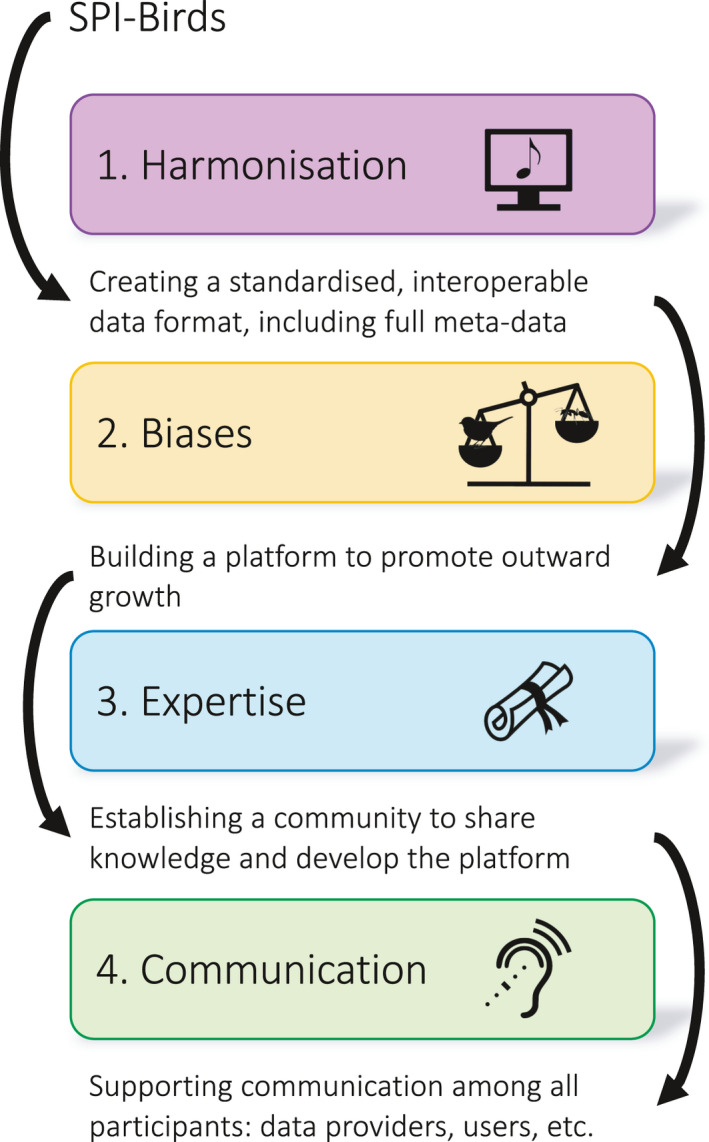
Four key challenges in the era of open data in ecology, and how the SPI‐Birds database (Culina et al., 2021 *)* has developed an effective platform to navigate these challenges

Despite the great progress made in the last decade in open data in ecology, one should not get too comfortable. The open, big data landscape that is starting to emerge in ecology brings new challenges that may test more traditional ecological mindsets (Hampton et al., 2013). Here, we discuss four of these challenges, namely regarding (1) harmonisation, (2) biases, (3) expertise and (4) communication (Figure 1). For each, we provide examples of how and why the challenge arises and how the framework employed by Culina et al. (2021) navigates them using FAIR principles as a model for future efforts in the era of open data.

## 1 CHALLENGE 1. HARMONISING OPEN DATA

Different datasets, even when collected strictly within the same sub‐field of ecology (e.g. animal population ecology), can differ vastly. For instance, ornithologists refer to the term ‘recruitment’ as the age at which an individual first reproduces (J.D. Lebreton, pers. comm. 2015; B. Sheldon, pers. comm. 2021), whereas plant ecologists refer to it as the germination of a seedling (Harper, 1977). Thus, it is strongly advised to harmonise (i.e. standardise and homogenise) data from various sources, or databases that house data from different researchers and sub‐disciplines, before the proposed analyses are conducted (Nadrowski et al., 2013) so that they are Interoperable and Reusable. Culina et al. (2021) navigate this through an interoperability pipeline and develop standardised formats for data such as the breeding season in the SPI‐bird data hub (Figure 1). Furthermore, database curators invest significant efforts and time harmonising data and complementing them with metadata, as well as creating thesaurus to help users navigate their rich platforms (e.g. Garnier et al., 2017; Pey et al., 2014). However, sometimes the information detailed in the original sources, such as MSc/PhD thesis, grey reports, peer‐review publications in different languages, does not allow for this task to be performed satisfactorily. When the harmonisation of data is incomplete, users of databases may benefit from the warnings and errors identified by database curators. For instance, in SPI‐bird (Culina et al., 2021), there are *standard quality checks*, and warnings are explicitly noted as values that are uncommon or unusual, while ‘likely errors’ are flagged as seemingly impossible values. It is important to note that the ultimate responsibility to correctly conduct an analysis with open‐access ecological databases remains with the user. Just because one *can* run an analysis with all the data at one's disposal, it does not mean one *should* do so.

## 2 CHALLENGE 2. BIASES IN OPEN DATA

Naturally, the search for broad global patterns in ecology will only be as robust as the data that analyses are based on. Many global ecological datasets are taxonomically biased towards mammals and birds. In the case of long‐term animal ecology datasets, a significant proportion of studies and databases are well represented primarily in areas of the planet with low biodiversity (Titley et al., 2017), or in areas that are actually least vulnerable to climate change (Paniw et al., 2021). Likewise, most terrestrial biodiversity is found in countries with low GDP, for which fewer data exist relative to countries with higher GDP. Like many initiatives, Culina et al. (2021) also display these geographic biases. Instead, the present study takes the approach of creating a framework and standards for the ‘well‐defined’ community (primarily in northern Europe) that acts as a platform for global efforts (Figure 1).

We propose several ways that ecologists may navigate this challenge. As a minimum, ecologists using open‐access data in ecology to search for global patterns must be aware of (and account for, where possible) geographic and taxonomic biases, contextualising findings rather than making blanket statements about findings occurring ‘worldwide’. Likewise, phylogenetic approaches offer numerous tools to impute missing data following patterns of phylogenetic inertia—but one needs to be aware of which tools fit the job better (Gallagher et al., 2020). Finally, cross‐matching algorithms to improve the overlap of interoperable databases can drastically increase the analytical power (Pennell et al., 2016). Ultimately however, greater international efforts are needed to increase the coverage of global biodiversity data in under‐represented countries. In this regard, the application of conservation prioritisation in data‐poor countries to expedite ecological data collection is a promising avenue of progress (El‐Gabbas et al., 2020; Kujala et al., 2018). Furthermore, the development of lasting partnerships between researchers in high‐income and low‐income countries to build capacity is required to even biases in ecological data archiving (Donhauser & Shaw, 2019).

## 3 CHALLENGE 3. EXPERTISE IN OPEN DATA

There is also a need to acquire the necessary expertise in the field to harness the full potential of the data. The multitude of records made available by, in this case, the SPI‐Bird data hub contain great potential. However, the large volumes of data cannot be a substitute for the invaluable ornithological expertise of the researchers who collected the data, nor the quantitative skills of researchers used to analyse them. Unfortunately, this kind of expertise also tends to be geographically clustered in countries with high GDP and relatively low biodiversity. Culina et al.'s approach to this challenge is to nurture an active community in which researchers and data users are engaged with one another and building skills through workshops/meetings (Figure 1). In our experience, this approach is essential for the effective implementation of large‐scale open‐data platforms that overcome geographic and sociopolitical biases. At COMPADRE (Salguero‐Gómez et al., 2015) and COMADRE (Salguero‐Gomez et al., 2016), for instance, we have run workshops in three different languages and in four continents on over 30 occasions, and we have now adopted a strategy where we prioritise attendance of researchers from low‐income countries.

## 4 CHALLENGE 4. IMPROVING COMMUNICATION OF THE OPEN‐DATA COMMUNITY

The era of big data in ecology is being support by a community composed of—at least—four different entities: data contributors, data curators, funding agencies and journals/societies. These entities risk failure of the whole enterprise if they do not adequately engage with each another. As such, communication and trust between them is critical. For instance, one of the main reasons that researchers may choose not to share data and contribute them to open‐access databases is the risk of being scooped (Laine, 2017). This reticence to share data can prevail even though research has shown that researchers who publish second still end up getting a substantial portion of the recognition (Callaway, 2019). A way that open‐data curators can support data contributors to overcome this initial concern is by offering an embargo period (something that we do in COMPADRE and COMADRE, but of which <1% of contributors request), or the possibility of making their data accessible (not open access) on the condition that they be offered co‐authorship. SPI‐Birds (Culina et al., 2021) partly follow the latter model, but with a minimal percentage of their total data (Figure 1). As a minimal requirement, SPI‐Birds users must explicitly acknowledge any data owners and funding sources of the raw data (stored in meta‐data). This not only improves communication in the community but also makes the raw data more findable in the future.

Database curators should make sure that credit be placed where it is due. Requesting that the original paper introducing a given database be cited when the database is used seems logical. However, what is even more logical—as well as fair and F.A.I.R.—is to request the individual contributing authors be cited too. This action to ensure appropriate accreditation may be hard to implement due to (1) the lack of database infrastructure to replicate a subset of citations in the final analysis and/or (2) the lack of space in journal prints to accommodate the potentially hundreds of the citations. For the former, some databases have already developed the functionality to provide database users with a citation summary of the data they have downloaded. For the latter, the move by many journals and societies from printed version to online only means that price‐per‐page is no longer a limitation to citation counts (Fox et al., 2016). In this way, data contributing researchers can benefit from other users utilising their data.

## 5 FINAL REMARKS

Noah's ecological data ark is beginning to get crowded. However, ecologists, data curators, funding agencies, journals and ecological societies need to adapt their mindsets, infrastructures and approaches to fill this ark faster, with fewer biases, and more efficiently. A more coordinated effort between data contributors, curators, users, journals and societies will result in much‐needed interoperability. Culina et al. (2021) is a testament to a new way of interaction, one that promotes FAIR principles to overcome these challenges and actively promotes international collaboration. Ultimately, the inherent value of SPI‐Birds (Culina et al., 2021) will grow exponentially when considered in conjunction with, for instance, the long‐term trends of insects on which birds depend (via InsectChange; Van Klink et al., 2021), human influence (via the Human Footprint Database; Venter et al., 2016) and climatic patterns (via CHELSA; Karger et al., 2017). The promise of big, open‐access data in ecology is huge. We must endeavour, as a community, to deliver it.

## CONFLICT OF INTEREST

The authors declare no conflicts of interest.

## AUTHORS' CONTRIBUTIONS

R.S.‐G. conceived the ideas and wrote the first version of the manuscript; J.J. created the figure; J.J. and S.J.L.G. contributed critically to the drafts and gave final approval for publication.

## References

[jane13567-bib-0001] Aplin, L. M. , Farine, D. R. , Morand‐Ferron, J. , Cockburn, A. , Thornton, A. , & Sheldon, B. C. (2015). Experimentally induced innovations lead to persistent culture via conformity in wild birds. Nature, 518, 538–541. 10.1038/nature13998 25470065 PMC4344839

[jane13567-bib-0002] British Ecological Society . (2016). Open data in the British Ecological Society. Retrieved from https://www.britishecologicalsociety.org/open‐data‐in‐the‐british‐ecological‐society‐journals/

[jane13567-bib-0003] Callaway, E. (2019). Scooped in science? Relax, credit will come your way. Nature, 575(7784), 576–577. 10.1038/d41586-019-03648-4 31776508

[jane13567-bib-0004] Clutton‐Brock, T. , & Sheldon, B. C. (2010). Individuals and populations: The role of long‐term, individual‐based studies of animals in ecology and evolutionary biology. Trends in Ecology & Evolution, 25, 562–573. 10.1016/j.tree.2010.08.002 20828863

[jane13567-bib-0005] Culina, A. , Adriaensen, F. , Bailey, L. D. , Burgess, M. D. , Charmantier, A. , Cole, E. F. , Eeva, T. , Matthysen, E. , Nater, C. R. , Sheldon, B. C. , Saether, B. E. , Vriend, S. J. G. , Zajkova, Z. , Adamik, P. , Aplin, L. M. , Angulo, E. , Artemyev, A. , Barba, E. , Barisic, S. , … Visser, M. E. (2021). Connecting the data landscape of long‐term ecological studies: The SPI‐Birds data hub. Journal of Animal Ecology. 10.1111/1365-2656.13388 PMC8518542 33205462

[jane13567-bib-0006] Donhauser, J. , & Shaw, J. (2019). Knowledge transfer in theoretical ecology: Implications for incommensurability, voluntarism, and pluralism. Studies in History and Philosophy of Science, 77, 11–20. 10.1016/j.shpsa.2018.06.011 31701874

[jane13567-bib-0007] El‐Gabbas, A. , Gilbert, F. , & Dormann, C. F. (2020). Spatial conservation prioritisation in data‐poor countries: A quantitative sensitivity analysis using multiple taxa. BMC Ecology, 20, 35. 10.1186/s12898-020-00305-7 32590973 PMC7318458

[jane13567-bib-0008] Farley, S. S. , Dawson, A. , Goring, S. J. , & Williams, J. W. (2018). Situating ecology as a big‐data science: Current advances, challenges, and solutions. BioScience, 68, 563–576. 10.1093/biosci/biy068

[jane13567-bib-0009] Firth, J. A. , Cole, E. F. , Ioannou, C. C. , Quinn, J. L. , Aplin, L. M. , Culina, A. , McMahon, K. , & Sheldon, B. C. (2018). Personality shapes pair bonding in a wild bird social system. Nature Ecology & Evolution, 2, 1696–1699. 10.1038/s41559-018-0670-8 30275466 PMC6217997

[jane13567-bib-0010] Fox, C. W. , Paine, C. E. T. , & Sauterey, B. (2016). Citations increase with manuscript length, author number, and references cited in ecology journals. Ecology and Evolution, 6, 7717–7726. 10.1002/ece3.2505 30128123 PMC6093155

[jane13567-bib-0011] Gallagher, R. V. , Falster, D. S. , Maitner, B. S. , Salguero‐Gomez, R. , Vandvik, V. , Pearse, W. D. , Schneider, F. D. , Kattge, J. , Poelen, J. H. , Madin, J. S. , Ankenbrand, M. J. , Penone, C. , Feng, X. , Adams, V. M. , Alroy, J. , Andrew, S. C. , Balk, M. A. , Bland, L. M. , Boyle, B. L. , … Enquist, B. J. (2020). Open Science principles for accelerating trait‐based science across the Tree of Life (vol. 4, p. 294, 2019). Nature Ecology & Evolution, 4, 662. 32066887 10.1038/s41559-020-1109-6

[jane13567-bib-0012] Garnier, E. , Stahl, U. , Laporte, M. A. , Kattge, J. , Mougenot, I. , Kuhn, I. , Laporte, B. , Amiaud, B. , Ahrestani, F. S. , Bonisch, G. , Bunker, D. E. , Cornelissen, J. H. C. , Diaz, S. , Enquist, B. J. , Gachet, S. , Jaureguiberry, P. , Kleyer, M. , Lavorel, S. , Maicher, L. , … Klotz, S. (2017). Towards a thesaurus of plant characteristics: An ecological contribution. Journal of Ecology, 105, 298–309. 10.1111/1365-2745.12698

[jane13567-bib-0013] Griffiths, J. I. , Childs, D. Z. , Bassar, R. D. , Coulson, T. , Reznick, D. N. , & Rees, M. (2020). Individual differences determine the strength of interactions. Proceedings of the National Academy of Sciences of the United States of America, 117, 17068–17073. 32631995 10.1073/pnas.2000635117 PMC7382284

[jane13567-bib-0014] Hampton, S. E. , Strasser, C. A. , Tewksbury, J. J. , Gram, W. K. , Budden, A. E. , Batcheller, A. L. , Duke, C. S. , & Porter, J. H. (2013). Big data and the future of ecology. Frontiers in Ecology and the Environment, 11, 156–162. 10.1890/120103

[jane13567-bib-0015] Harper, J. L. (1977). Population biology of plants. Academic Press.

[jane13567-bib-0016] Karger, D. N. , Conrad, O. , Bohner, J. , Kawohl, T. , Kreft, H. , Soria‐Auza, R. W. , Zimmermann, N. E. , Linder, H. P. , & Kessler, M. (2017). Data descriptor: Climatologies at high resolution for the earth's land surface areas. Scientific Data, 4, 170122. 10.1038/sdata.2017.122 28872642 PMC5584396

[jane13567-bib-0017] Kujala, H. , Lahoz‐Monfort, J. J. , Elith, J. , & Moilanen, A. (2018). Not all data are equal: Influence of data type and amount in spatial conservation prioritisation. Methods in Ecology and Evolution, 9, 2249–2261. 10.1111/2041-210X.13084

[jane13567-bib-0018] Laine, H. (2017). Afraid of scooping – Case study on researchers strategies against fear of scooping in the context of open science. Data Science Journal, 16, 29.

[jane13567-bib-0019] Mills, J. A. , Teplitsky, C. , Arroyo, B. , Charmantier, A. , Becker, P. H. , Birkhead, T. R. , Bize, P. , Blumstein, D. T. , Bonenfant, C. , Boutin, S. , Bushuev, A. , Cam, E. , Cockburn, A. , Cote, S. D. , Coulson, J. C. , Daunt, F. , Dingemanse, N. J. , Doligez, B. , Drummond, H. , … Zedrosser, A. (2015). Archiving primary data: Solutions for long‐term studies. Trends in Ecology & Evolution, 30, 581–589. 10.1016/j.tree.2015.07.006 26411615

[jane13567-bib-0020] Nadrowski, K. , Ratcliffe, S. , Bonisch, G. , Bruelheide, H. , Kattge, J. , Liu, X. J. , Maicher, L. , Mi, X. C. , Prilop, M. , Seifarth, D. , Welter, K. , Windisch, S. , & Wirth, C. (2013). Harmonizing, annotating and sharing data in biodiversityecosystem functioning research. Methods in Ecology and Evolution, 4, 201–205. 10.1111/2041-210x.12009

[jane13567-bib-0021] Paniw, M. , James, T. D. , Ruth Archer, C. , Römer, G. , Levin, S. , Compagnoni, A. , Che‐Castaldo, J. , Bennett, J. M. , Mooney, A. , Childs, D. Z. , Ozgul, A. , Jones, O. R. , Burns, J. H. , Beckerman, A. P. , Patwary, A. , Sanchez‐Gassen, N. , Knight, T. M. , & Salguero‐Gómez, R. (2021). The myriad of complex demographic responses of terrestrial mammals to climate change and gaps of knowledge: A global analysis. Journal of Animal Ecology, 90, 1398–1407. 10.1111/1365-2656.13467 33825186

[jane13567-bib-0022] Paniw, M. , Maag, N. , Cozzi, G. , Clutton‐Brock, T. , & Ozgul, A. (2019). Life history responses of meerkats to seasonal changes in extreme environments. Science, 363, 631–635. 10.1126/science.aau5905 30733418

[jane13567-bib-0023] Pennell, M. W. , FitzJohn, R. G. , & Cornwell, W. K. (2016). A simple approach for maximizing the overlap of phylogenetic and comparative data. Methods in Ecology and Evolution, 7, 751–758. 10.1111/2041-210X.12517 PMC4957270 27499839

[jane13567-bib-0024] Pey, B. , Laporte, M. A. , Nahmani, J. , Auclerc, A. , Capowiez, Y. , Caro, G. , Cluzeau, D. , Cortet, J. , Decaens, T. , Dubs, F. , Joimel, S. , Guernion, M. , Briard, C. , Grumiaux, F. , Laporte, B. , Pasquet, A. , Pelosi, C. , Pernin, C. , Ponge, J. F. , … Hedde, M. (2014). A Thesaurus for soil invertebrate trait‐based approaches. PLoS ONE, 9, e108985. 10.1371/journal.pone.0108985 25310431 PMC4195653

[jane13567-bib-0025] Salguero‐Gomez, R. , Jones, O. R. , Archer, C. R. , Bein, C. , de Buhr, H. , Farack, C. , Gottschalk, F. , Hartmann, A. , Henning, A. , Hoppe, G. , Romer, G. , Ruoff, T. , Sommer, V. , Wille, J. , Voigt, J. , Zeh, S. , Vieregg, D. , Buckley, Y. M. , Che‐Castaldo, J. , … Vaupel, J. W. (2016). COMADRE: A global data base of animal demography. Journal of Animal Ecology, 85, 371–384. 10.1111/1365-2656.12482 PMC4819704 26814420

[jane13567-bib-0026] Salguero‐Gómez, R. , Jones, O. J. , Archer, C. R. , Buckley, Y. M. , Che‐Castaldo, J. P. , Caswell, H. , Hodgson, D. J. , Scheuerlein, A. , Conde, D. A. , Brinks, E. , Farack, C. , Gottschalk, F. , Hartmann, A. , Henning, A. , Hoppe, G. , Römer, G. , Runge, J. , Ruoff, T. , Wille, J. , … Vaupel, J. W. (2015). The COMPADRE Plant Matrix Database: An open online repository for plant demography. Journal of Ecology, 103, 202–208.

[jane13567-bib-0027] Titley, M. A. , Snaddon, J. L. , & Turner, E. C. (2017). Scientific research on animal biodiversity is systematically biased towards vertebrates and temperate regions. PLoS ONE, 12, e0189577. 10.1371/journal.pone.0189577 29240835 PMC5730207

[jane13567-bib-0028] Van Klink, R. , Bowler, D. E. , Driessen, M. M. , Ernest, S. K. M. , Gentile, A. , Gilbert, F. , Gongalsky, K. B. , Owen, J. , Pe'er, G. , Pe'er, I. , Resh, V. H. , Rochlin, I. , Schuch, S. , Swengel, A. E. , Swengel, S. R. , Valone, T. J. , Vermeulen, R. , Wepprich, T. , Wiedmann, J. L. , & Chase, J. M. (2021). InsectChange: A global database of temporal changes in insect and arachnid assemblages. Ecology, 102, e03354. 33797755 10.1002/ecy.3354

[jane13567-bib-0029] Venter, O. , Sanderson, E. W. , Magrach, A. , Allan, J. R. , Beher, J. , Jones, K. R. , Possingham, H. P. , Laurance, W. F. , Wood, P. , Fekete, B. M. , Levy, M. A. , & Watson, J. E. M. (2016). Sixteen years of change in the global terrestrial human footprint and implications for biodiversity conservation. Nature Communications, 7, 11. 10.1038/ncomms12558 PMC4996975 27552116

[jane13567-bib-0030] Wilkinson, M. D. , Dumontier, M. , Aalbersberg, I. J. J. , Appleton, G. , Axton, M. , Baak, A. , Blomberg, N. , Boiten, J.‐W. , da Silva Santos, L. B. , Bourne, P. E. , Bouwman, J. , Brookes, A. J. , Clark, T. , Crosas, M. , Dillo, I. , Dumon, O. , Edmunds, S. , Evelo, C. T. , Finkers, R. , … Mons, B. (2016). Comment: The FAIR Guiding Principles for scientific data management and stewardship. Scientific Data, 3, 160018. 10.1038/sdata.2016.18 26978244 PMC4792175

[jane13567-bib-0031] Wilson, E. O. (2017). Biodiversity research requires more boots on the ground. Nature Ecology & Evolution, 1, 1590–1591. 10.1038/s41559-017-0360-y 29066811

